# Flavescence dorée phytoplasma enters insect cells by a clathrin-mediated endocytosis allowing infection of its insect vector

**DOI:** 10.1038/s41598-023-29341-1

**Published:** 2023-02-07

**Authors:** Nathalie Arricau-Bouvery, Marie-Pierre Dubrana, Francesca Canuto, Sybille Duret, Lysiane Brocard, Stéphane Claverol, Sylvie Malembic-Maher, Xavier Foissac

**Affiliations:** 1 Univ. Bordeaux, INRAE, Biologie du Fruit et Pathologie, UMR 1332, 33140 Villenave d’Ornon, France; 2 Univ. Bordeaux, CNRS, INSERM, Bordeaux Imaging Center, BIC, UAR 3420, US 4, 33140 Villenave d’Ornon, France; 3 Plateforme Protéome, Univ. Bordeaux, Bordeaux, France

**Keywords:** Bacteria, Cellular microbiology, Pathogens

## Abstract

To perform its propagative and circulative cycle into its insect vector, the flavescence dorée phytoplasma invades different cell types. Clathrin-mediated endocytosis is used by a wide range of bacteria to infect eukaryote cells. Among the insect proteins interacting with the phytoplasma adhesin VmpA, we identified the adaptor protein complex AP-1 and AP-2 suggesting that phytoplasmas could enter the insect cells via clathrin-mediated endocytosis. By infection assays of insect cells in culture, we showed that phytoplasmas entry into *Drosophila* S2 cells was more efficient than infection of the Euva cell line developed from the insect vector *Euscelidius variegatus*. Chlorpromazine, cytochalasin D and knockdown of clathrin heavy chain (chc) gene expression using RNA interference inhibited entry of phytoplasmas into S2 cells. During invasion of S2 cells, phytoplasmas were observed very closed to recombinant GFP-labelled clathrin light chain. To verify the role of clathrin in the insect colonization by phytoplasmas, RNAi was performed via artificial feeding of chc dsRNA by the vector *E. variegatus*. This decreased the expression of chc gene in the midgut and heads of *E. variegatus*. The chc lower expression correlated to a decreased of midgut and salivary gland cells colonization after the insects had ingested phytoplasmas from infected plants. In conclusion, results indicate that clathrin is important for the FD phytoplasma to enter insect cells and colonize its insect vector.

## Introduction

The Flavescence dorée phytoplasma (FDP) is a quarantine pest affecting European grapevine. It is responsible of high economic losses due to reduction of yield, compulsory surveillance of the vineyards, removal of infected plants and repeated sprays of insecticides^1,2^. The causal agent responsible for FD is a small wall-less bacterium belonging to the class *Mollicutes* and named phytoplasma^3–5^. This bacterium resisted so far to axenic cultivation in cell-free media and thus cannot be genetically engineered. It is transmitted in a persistent manner from grapevine to grapevine by the leafhopper *Scaphoideus titanus*^6–8^. In addition to its natural transmission by *S*. *titanus*, FDP can also be experimentally transmitted from broad bean to broad bean by the leafhopper *Euscelidius variegatus*^9^. To be transmitted to plant, phytoplasmas have to perform a propagative and circulative cycle into its insect vector^10–13^. For this, phytoplasmas invade insect cells of different organs in which they multiply. Phytoplasmas must initially colonize and cross the midgut epithelium, reach the haemolymph, invade several tissues including the salivary gland cells to be finally injected back to the plant phloem sap through infected saliva^14^. Adhesion of phytoplasmas to insect cells is a prerequisite for their internalization into the vector cells. Proteins exposed to the phytoplasma surface such as Amp (Antigenic membrane protein) or Imp (Immunodominant membrane protein) were found to interact with different insect vector proteins that belong to the cytoskeleton or ATP synthase^15–20^. Due to the intracellular location of these targeted host cell structures, these interactions should not be implicated in the primary adhesion of phytoplasmas to the insect cells but instead, should take place during the subsequent intracellular trafficking of phytoplasmas. In the case of FD phytoplasmas, the ecological adaptation of FDP to different insect vectors correlate with the variability of variable membrane protein vmpA and vmpB protein sequences^21^. VmpA mediates adhesion to insect cells through binding the glycoproteins present in the perimicrovillar membrane of the midgut and at the surface of the salivary gland cells^22,23^. However, the mechanism by which phytoplasmas enter the insect cells is not known yet but it may involve receptor-mediated endocytosis.

Bacteria enter eukaryotic nonphagocytic cells using different ways like “zipper” and “trigger” mechanisms. While “triggering” bacteria gain access through injected effector proteins, in the zipper procedure, the bacterial surface proteins, also called adhesins, directly bind to host cellular receptors and initiates a signalling cascade of endocytic pathways, including clathrin-mediated endocytosis^24,25^. Glycoproteins present at the surface of eukaryotic cells can serve as receptors for the endocytosis of bacteria into the cells via a clathrin-dependant mechanism. For example, the adhesin FimH of uropathogenic *Escherichia coli* recognizes mannose and mannose-containing glycoproteins, including integrin, and the glycoprotein E-cadherin is the receptor for InlA of *L*. *monocytogenes* allowing entry of *E*. *coli* and *L*. *monocytogenes* into eukaryotic cell via clathrin-mediated endocytosis^26–28^. The adaptor proteins (AP) that interact with the receptors and clathrin and are required for the internalization of *Listeria monocytogenes* into mammalian cells are the proteins Dab2 and AP-1^29^. In the case of *Brucella abortus*, not only the entry into host cells requires clathrin but also the early intracellular trafficking and survival of the bacteria into the eukaryotic cell^30^. *Wolbachia* spp. also uses clathrin-mediated endocytosis for entry into drosophila insect cells in culture as demonstrated during horizontal cell-to-cell transfer^31^. It has also been shown that the entry of many viruses into insect cells is mediated via clathrin. For example, all four serotypes of the dengue virus enter the mosquito cells C6/36 via the clathrin-dependent endocytosis^32,33^. In the case of insect-transmitted viruses pathogenic for plants, the TYLCV (tomato yellow leaf curl virus) begomovirus enters the midgut cells of the whitefly *Bemisia tabaci* using clathrin-mediated endocytosis^34^, and the RDV (rice dwarf virus) phytoreoviru*s* uses clathrin-mediated endocytosis to infect the cultured cells of its insect vector *Nephotettix cincticeps*^35^. However, the caveolae-dependent endocytosis^36^ is also used by other bacteria such as group B streptococci, *Treponema pallidum* or *Campylobacter jejuni*, to enter their host cells and escape the acidic condition of lysosomes^37–39^.

In this report, we identified by mass spectrometry adaptor proteins among the protein complexes that interacted with the FD phytoplasma adhesin VmpA. Considering this result and that VmpA binds glycoconjugates present at the surface of insect cells, we hypothesize that clathrin could be implicated into the invasion of insect cells by phytoplasmas. We thus investigated if entry of phytoplasmas in host cells was mediated by a clathrin-mediated endocytosis using *in cellulo* and in vivo experiments.

## Results

### Identification of the insect proteins interacting with VmpA

**Figure 1:**
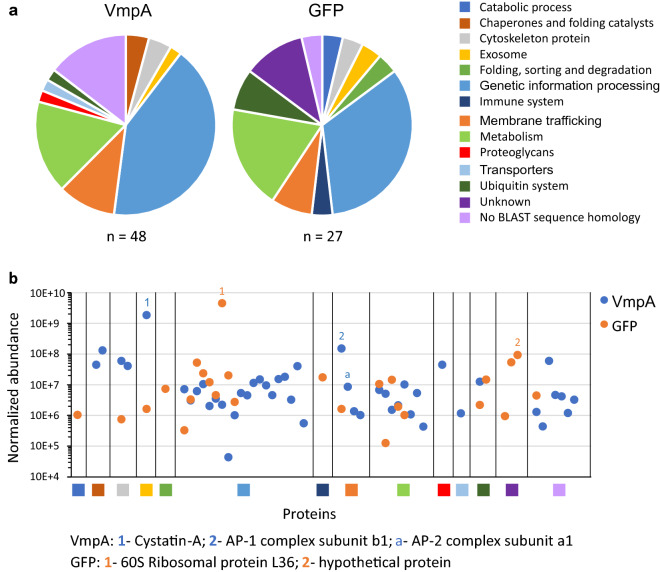
Distribution of proteins interacting with VmpA or GFP after pull-down purification and LS-MS/MS identification. The proteins that significantly interacted more with VmpA than with GFP (VmpA) or more with GFP than with VmpA (GFP) were classified in function of the KEGG BRITE classification (**a**) or represented in function of their normalized abundance (**b**). The two proteins with the highest abundance identified for each condition are indicated in the graph and named under the graph.

To capture the insect proteins interacting with VmpA, pull down assays were attempted with intestinal or salivary gland proteins of *E. variegatus*. Unfortunately, very few proteins were detected after protein extractions from midguts. We thus performed pull down assays mixing salivary gland proteins with magnetic beads coated with VmpA, or GFP as control. After identification of the peptides produced by LC–MS/MS, the proteins that interacted with the proteins VmpA and GFP were classified according to the biological process they are involved in (Fig. 1a). A total of 48 different proteins that significantly interacted more with VmpA-His_6_-coated beads than with GFP-coated beads were identified (Table S1). Only 27 different proteins interacted more with GFP-coated beads than with VmpA-His_6_-coated beads. The percentages of the proteins grouped in the different biological processes were very similar between VmpA and GFP pull-down. Exceptions were found for the proteins with no BLAST sequence homology, 15% interacted with VmpA-His_6_-coated beads compared to 5% that interacted with GFP-coated beads, and more proteins that interacted with GFP-coated beads belonged to unknown proteins (14%) compared to proteins that interacted with VmpA-coated beads (0%). The proteins associated with membranes were also three-fold represented in proteins interacting with VmpA in comparison with GFP (proteins grouped in Membrane trafficking, Proteoglycans and Transporters). Among the proteins preferentially interacting with the protein VmpA the two most abundant proteins were cystatin and AP-1 adaptor protein complex (Fig. 1b). Moreover, the AP-2 adaptor protein complex that also interact with clathrin was found in the proteins that interacted more with VmpA than the GFP protein. The two most abundant proteins that interacted with the GFP protein were the 60S ribosomal protein L36 and a hypothetical protein. We thus tested the hypothesis that FDP could enter insect cells using clathrin-mediated endocytosis.

### Infection of Euva-12 and S2 cells by flavescence dorée phytoplasma

**Figure 2:**
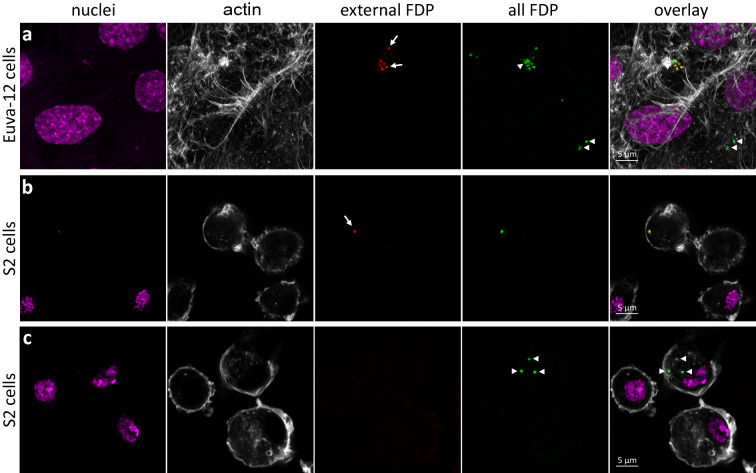
Observation of Euva-12 and S2 cells 3 days post-infection with FD phytoplasmas (FDP). (**a**) Euva-12 infected with FDP. Each picture represents the maximum projection image of 5 z-planes. (**b**, **c**), S2 cells infected with FDP showing an external phytoplasma (**b**), and internalized phytoplasmas (**c**) in which the pictures represent the maximum projection image of 2 z-planes. The nuclei were stained with DAPI and coloured in magenta; the actin filaments were stained with Alexa 568-phalloidin and coloured in grey; external phytoplasmas were stained with Alexa 633 and coloured in red; after permeabilization of the infected cells, both internalized and external phytoplasmas were stained with Alexa 488 and coloured in green. Arrowheads indicate internalized phytoplasmas and arrows external phytoplasmas.

We first evaluated the ability of the FD phytoplasmas to enter the *E. variegatus* Euva-12 cells in culture. The phytoplasma inoculum was prepared from *E. variegatus* salivary glands as phytoplasmas multiply at high rate within several cell types of this organ^23^. We thus used crushed salivary glands of phytoplasma-infected *E. variegatus* to inoculate the Euva-12 cells. Three days post inoculation, phytoplasmas were observed both at the surface of the Euva-12 cells and internalized into the cells as respectively indicated by the yellow and green colour on overlay picture (Fig. 2a). However, with this cell line, the percentage of internalized phytoplasmas did not exceed 10%. We tested several times of infection, up to 21 days, but the percentage of intracellular phytoplasmas did not increase. We therefore used the S2 cell line of *Drosophila* that are phagocytes and would internalized more phytoplasmas than Euva-12 cells.

The S2 cells were infected in the same conditions. One to three phytoplasmas were generally observed per infected cell (Fig. 2b,c). Both extra- and intra-cellular phytoplasmas were observed, but the percentage of intracellular phytoplasmas was 36% (± 7) in regard to the total number of phytoplasmas observed per field. This higher number of internalized phytoplasmas prompted us to use the S2 cells to analyse the mechanism used by the FD phytoplasma to enter into insect cells.

### Inhibition of FD phytoplasma entry into S2 cells by cytochalasin D and chlorpromazine

S2 cells were used to test different inhibitors of endocytosis that were chlorpromazine (CPZ), an inhibitor of the clathrin-mediated endocytosis, cytochalasin D (Cyto D), which inhibits the polymerization of actin filaments and thus inhibits different endocytosis pathways, and nystatin (Nyst), a cholesterol sequestering agent and inhibitor of caveolin-dependant endocytosis. We previously verified that these inhibitors did not affect the viability of the cells. The percentages of viable S2 cells was similar whatever the drug and the concentration used, 10 or 30 µM (Table 1). We thus used both concentrations before infection of S2 cells with FD phytoplasmas.

**Table 1:** Viability of S2 cells incubated with chlorpromazine (CPZ), cytochalasin D (Cyto D) and nystatin (Nyst) observed one day post treatment.

Inhibitor	Concentration (µM)	Viability
No	–	99.7
CPZ	10	98.4
	30	94.4
Cyto D	10	97.1
	30	93.7
Nyst	10	99.6
	30	98.3

**Figure 3:**
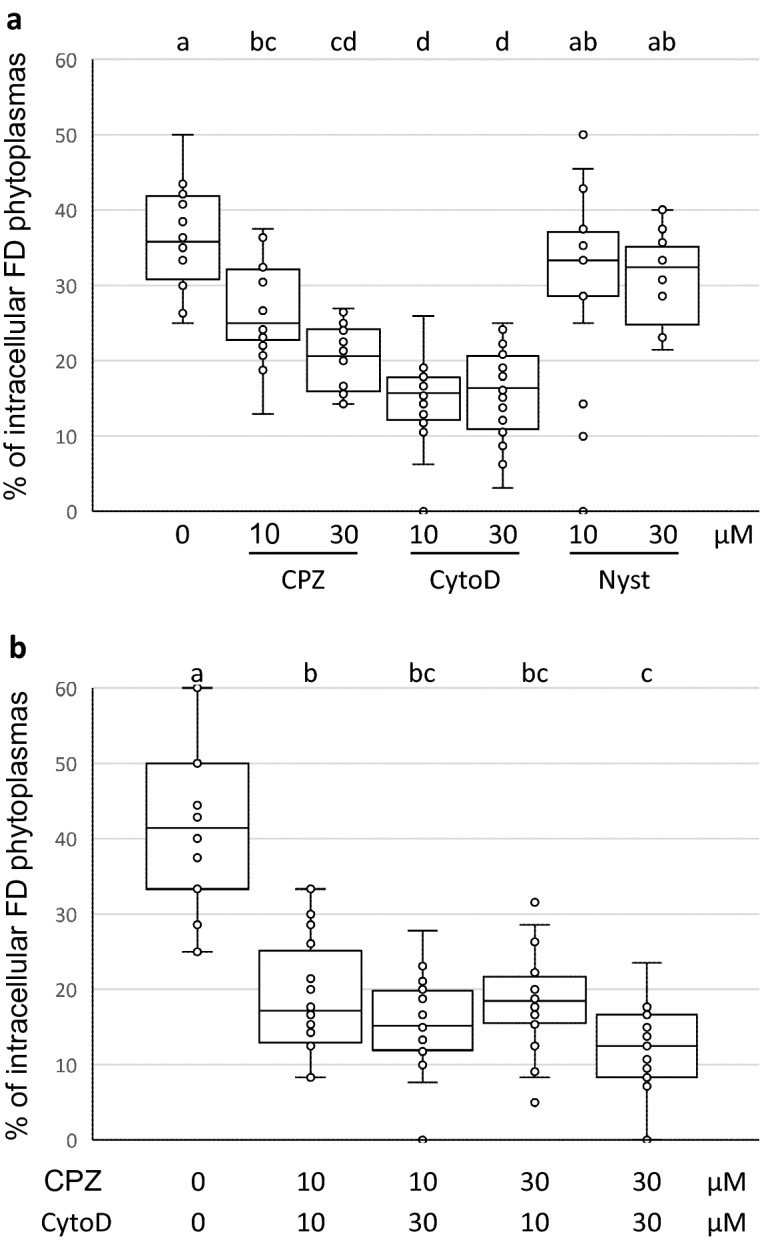
Internalization of FD phytoplasmas into S2 cells in presence of chlorpromazine (CPZ), cytochalasin D (CytoD) and nystatin (Nyst). Two concentrations (10 and 30 µM) were used for each inhibitor. Boxplots with different letters are significantly different under the ANOVA test of the R commander package of R software version 4.0.3^63^ (https://www.R-project.org). The normal distribution of the value was previously verified using the Shapiro–Wilk normality test of R software (R commander package).

On average, 51 (± 5) S2 cells and 18 (± 9) phytoplasmas were observed per field, 20 fields were photographed per condition, and three independent experiments were performed. The results show that the three inhibitors did not have the same effect on the internalization of the FDP by the S2 cells (Fig. 3a). Nyst had no significant effect whatever the concentration used, suggesting that entry of FD phytoplasmas was not associated with caveolae. A significant decrease of FDP internalization was observed with CPZ at the two concentrations tested. These results suggest that phytoplasmas enter S2 cells, and probably cells of its insect vectors, using clathrin-mediated endocytosis. Cyto D had a stronger effect than CPZ on the inhibition of FDP internalization suggesting that phytoplasmas could also enter via alternative pathways than clathrin-dependent endocytosis in S2 cells.

In order to see the cumulative effect of these two inhibitors, we treated the insect cells with both CPZ and Cyto D at the two concentrations. As soon as the smallest concentrations were used, we observed a strong inhibition of the entry of phytoplasmas into S2 cells (Fig. 3b). The inhibition factor was 1.4 with CPZ 10 µM alone in regard to the mean (Fig. 3a), and increased to 2.2 when Cyto D 10 µM was added to CPZ (Fig. 3b). Addition of CPZ 30 µM intensified the effect of Cyto D since 3.5 times less of internalized phytoplasmas in average were counted in presence of CPZ 30 µM plus Cyto D 30 µM (Fig. 3b) compared to 2.3 times less in presence of Cyto D 30 alone (Fig. 3a).

### Inhibition of FD phytoplasma entry into S2 cells using RNA interference

**Figure 4:**
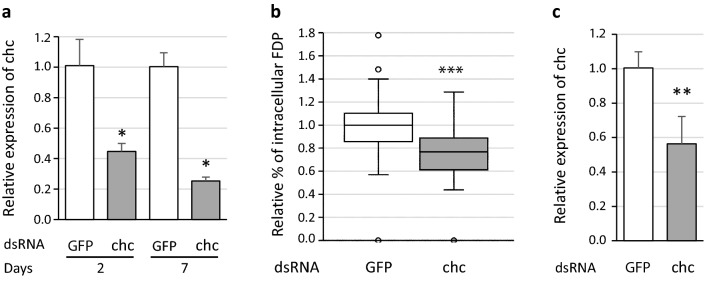
Internalization of FD phytoplasmas (FDP) into S2 cells in presence of dsRNA chc (clathrin heavy chain) and GFP. (**a**) S2 cells were incubated for 2 or 7 days with dsRNA GFP (control, white boxes) or dsRNA chc (grey boxes). Expression of the chc gene was measured regards to the reference genes glutathione S-transferase and elongator factor 1. *Indicates a significant difference with p = 0.0463 at 2 days and p = 0.0495 at 7 days under the Kruskal–Wallis rank sum test of the R commander package of R software version 4.0.3 (R: A language and Environment for statistical computing, R Core Team, R Foundation for Statistical Computing, Vienna, Austria, 2020, https://www.R-project.org). (**b**) internalization of FD phytoplasmas into S2 cells seven days after the cells were transfected with dsRNA GFP (white) or dsRNA chc (grey). ***Indicates a significant difference with p = 1.3 × 10–10 under the Kruskal–Wallis rank sum test. (**c**) control of RNAi efficiency into S2 cells transfected at the same time as cells infected in (**b**). The chc gene expression was measured regards to the reference genes glutathione S-transferase and elongator factor 1. **Indicates a significant difference with p = 0.00265 under the Kruskal–Wallis rank sum test.

Another way to inhibit the clathrin-mediated endocytosis is the use of RNA interference (RNAi) targeting the clathrin heavy chain (chc) gene expression. We thus transfected S2 cells with dsRNA homologs to the chc gene, and with GFP dsRNA as control. Total RNA was extracted at 2 and 7 days after transfection, and real time RT-PCR was used to quantify the effect of dsRNA on the expression of the chc gene. Similar results were obtained with the two reference genes glutathione S-transferase (GST) and elongator factor 1 (EF1) used (data unknown). The relative expression decreased from 2.3-fold at 2 days, to fourfold at 7 days when the S2 cells were transfected with chc dsRNA compared to GFP dsRNA (Fig. 4a). We thus infected S2 cells with FD phytoplasmas 7 days after dsRNA treatment. Transfection with dsRNA chc decreased the entry of phytoplasmas into S2 cells compared to cells transfected with dsRNA GFP (Fig. 4b). The relative expression of chc gene was also measured to show that RNAi had been efficient (Fig. 4c).

### Localization of FD phytoplasma and GFP-clathrin in S2 cells

**Figure 5:**
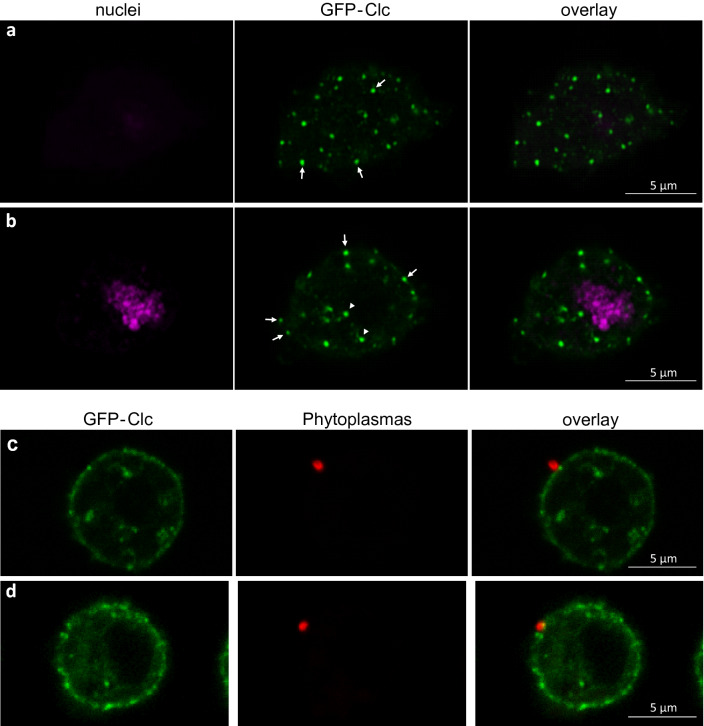
Observation of S2 cells expressing clathrin light chain tagged with GFP (GFP-Clc) and infected with FD phytoplasmas. (**a**, **b**) S2 cell expressing GFP-Clc and imaged at the surface of the cell (**a**) and inside the cell (**b**). Arrows indicate dots of GFP-Clc at the surface of the cells and arrowheads internal GFP-Clc dots. The nuclei were stained with DAPI and coloured in magenta; the GFP-Clc was coloured in green. (**c**, **d**), two different S2 cells expressing GFP-Clc and infected with FD phytoplasmas. The GFP-Clc was coloured in green, phytoplasmas were stained with Alexa 568 and coloured in red.

To co-localize phytoplasmas and clathrin in the S2 cells, we used clathrin light chain tagged with GFP (GFP-Clc) and anti-VmpA antibodies. In non-infected S2 cells, the GFP-Clc was localized near the surface of the S2 cells where small dots were clearly visible (Fig. 5a). Fluorescence was also detected in the cytoplasm with bright dots too (Fig. 5b). Such localization near the membrane was similar to that was observed in haemocytes extracted from clc-GFP *Drosophila* strain^40^. Before infecting S2 cells with phytoplasmas, we selected those expressing GFP-Clc using blasticidine resistance and flow cytometry to obtain a culture with about 80% of fluorescent cells. S2 cells expressing GFP-Clc were infected with FDP and observed 18 h post infection. In 70% of cases, phytoplasmas were observed at the surface of S2 cells closed to green fluorescent dots (Fig. 5c,d). This suggests that phytoplasmas can recruit these structures containing clathrin or induced nucleation of clathrin at the contact with insect cells.

### Inhibition of chc gene expression of *E. variegatus* using RNAi

To test the implication of the clathrin in the entry of phytoplasmas into insect vector cells in vivo, we infected by FDP *E. variegatus* in which the expression of clathrin heavy chain (chc) gene was affected by RNAi. We first tested the inhibition of the chc gene expression by ingestion of dsRNAs by *E. variegatus* using artificial feeding. As control, insects ingested GFP dsRNA before to be transferred on FDP infected broad beans for 7 days (acquisition access period, AAP) and then on healthy plants for latency period (LP).

After 2 days of ingestion of chc dsRNA, insects were placed onto FDP infected broad beans for 7 days of AAP or onto non-infected plants for 7 days to verify if the presence of phytoplasmas would change the insect RNAi efficiency. After 2 days of dsRNA ingestion, the survival rates were very similar between the insects that ingested GFP dsRNA (mean of 87%) and chc dsRNA (mean of 85%) whatever the experiment considered (Table 2). These rates decreased seven days after the insects were placed on broad beans and were 76% when *E. variegatus* ingested GFP dsRNA and then placed onto FDP infected broad beans, 73% and 77% when insects ingested chc dsRNA and then put onto FDP infected and non-infected broad beans, respectively. These survival rates were sufficient to test the effect of RNAi onto *E. variegatus* up to 30 days after the 2 days of dsRNA ingestion.

**Table 2:** Survival rates observed after two days of dsRNA ingestion by artificial feeding and 7 days after the insects were placed on FDP-infected broad beans or non-infected plants.

Experimentation		After 2 days of dsRNA ingestion		After 2 days of dsRNA ingestion + 7 days on broad bean		
Date	dsRNA	No of insects	Survival (%)	Plant latency	No of insects	Survival (%)
05/2021	GFP	114	92	FDP infected	105	92
	chc	238	94	FDP infected	111	81
				Non-infected	113	82
06/2021	GFP	150	93	FDP infected	130	62
	chc	300	85	FDP infected	120	63
				Non-infected	120	53
07/2021	GFP	150	84	FDP infected	120	73
	chc	300	83	FDP infected	120	55
				Non-infected	120	86
10/2021	GFP	154	81	FDP infected	117	78
	chc	297	77	FDP infected	106	92
				Non-infected	104	88

**Figure 6:**
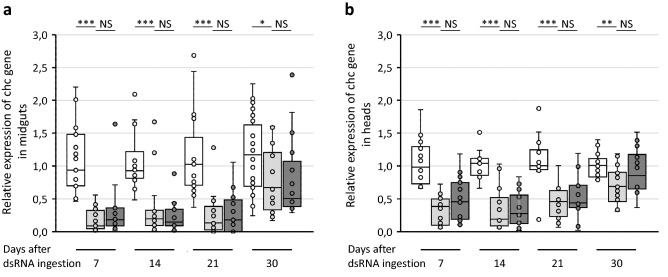
Expression of the clathrin heavy chain (chc) gene in the midguts (**a**) and in the heads (**b**) regards to the reference genes glutathione S-transferase and tubulin β. White boxes correspond to *E. variegatus* that ingested GFP dsRNA and acquired phytoplasmas on infected broad beans for seven days. Light grey boxes correspond to *E. variegatus* that ingested chc dsRNA and acquired phytoplasmas on infected broad beans for seven days and then placed onto non-infected broad beans. Dark grey boxes correspond to *E. variegatus* that ingested chc dsRNA and were then placed onto non-infected broad beans. *Indicates a significant difference with p < 0.05, **with p < 0.01, ***with p < 0.001 and NS indicates no significant difference under the Kruskal–Wallis rank sum test of the R commander package of R software version 4.0.3.

Seven, 14, 21 and 30 days after the 2 days of dsRNA ingestion, 10 insects per time point were dissected to separate the heads and the midguts. The heads and midguts of two insects were then pooled to have five samples per organ, experiment and time point. Total RNA was extracted and real time RT-PCR was used to quantify the effect of dsRNA ingestion on the expression of the chc gene. The relative expression level was calculated using the glutathione S-transferase (GST) and tubulin β (Tubβ) as reference genes. In the midgut, the chc dsRNA ingestion induced a fivefold reduction in chc mRNA levels at 7 days after ingestion of the chc dsRNA compared to the insects that ingested GFP dsRNAs (Fig. 6a). Then the decrease of chc mRNA levels was less important over the time-lapse but the chc mRNA levels remained statistically lower up to 30 days.

A decrease of chc mRNA level was also observed in the *E. variegatus* heads but was lightly less important than in the midguts of the insects. In this case, the chc dsRNA ingestion induced a 2.3-fold reduction in chc mRNA levels at 7 days after ingestion of the chc dsRNA compared to the insects that ingested GFP dsRNAs and 5.4-fold at 14 days (Fig. 6b). These differences remained statistically different at 30 days post-ingestion.

No statistically difference of the chc mRNA levels was found between the insects that had ingested chc dsRNA and then placed on FDP infected plants or non-infected plants, whatever the time point or the organ considered. These results implied that the phytoplasmas did not seem to decrease the RNAi response of *E. variegatus* in our conditions and that ingestion of chc dsRNAs had a time-dependent effect on the expression of the chc target gene both in the midgut and the head of insects.

### RNAi decreases the multiplication of FD phytoplasma in intestine and salivary gland cells

To see the effect of RNAi on phytoplasma colonization of *E. variegatus*, we quantified the FDP elongator factor Tu (tuf) mRNA among the mRNA extracted from midguts and heads of insects that ingested chc dsRNAs, or GFP dsRNAs as control. Considering that phytoplasmas multiply at a high rate in the salivary gland cells and weakly detected in the other organs of the head, we judged the presence of FD phytoplasmas in insect heads corresponded to the phytoplasmas present in the salivary glands. The percentage of insects in which phytoplasmas were detected was 100% at 7 days post ingestion whatever the dsRNA ingested.

**Figure 7:**
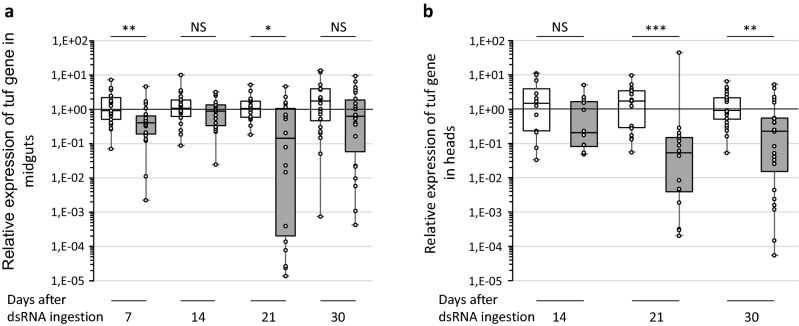
Expression of the phytoplasma elongator factor Tu (tuf) gene in the midguts (**a**) and in the salivary glands (**b**) regards to the reference genes glutathione S-transferase and tubulin β. White boxes correspond to *E. variegatus* that ingested GFP dsRNA and acquired phytoplasmas on infected broad beans for 7 days. Grey boxes correspond to *E. variegatus* that ingested chc dsRNA and acquired phytoplasmas on infected broad beans for seven days. The insects were then placed onto non-infected broad beans. *Indicates a significant difference with p < 0.05, **with p < 0.01, ***with p < 0.001 and NS indicates no significant difference under the Kruskal–Wallis rank sum test of the R commander package of R software version 4.0.3.

In the midgut, the relative quantities of tuf mRNAs was inferior in the insects that ingested chc dsRNAs than those which ingested GFP dsRNAs (Fig. 7a). These differences were significant at 7 days and 21 days post ingestion of dsRNA. The phytoplasma tuf mRNAs amounts were comprised between 1.7 and 7.6-fold lower over the time lapse studied. In the salivary gland cells, the number of tuf mRNAs was also lower in presence of chc dsRNA and these differences were significant at 21- and 30-days post ingestion (Fig. 7b). In this case, the quantities of tuf mRNAs decreased with 7.2-fold at 14 days, 33-fold at 21 days and fourfold at 31 days. These results show that inhibition of the *chc* gene expression reduced the multiplication of the phytoplasmas in its insect vector, both in the midgut and salivary gland cells.

## Discussion

Entry of viruses in insect cells using clathrin-mediated endocytosis seems to be widespread in insect vector of plant diseases. Clathrin is also a determinant factor for the infection of *Drosophila* S2 cells by the bacteria *Wolbachia*^31^. In our study and to our knowledge, the insect transmissible FDP is the second known example of a bacteria that uses clathrin to infect insect cells. Like in our case, CPZ decreased *Wolbachia* entry into S2 cells but did not inhibit it. We found similar results when clathrin expression was partially inhibited using RNAi. As measured by RT-PCR, the efficiency of RNAi was not as strong as for the first experiments on the mRNA chc decrease (Fig. 4c, 1.8-fold decrease). This could explain the slight but significant decrease of phytoplasma internalisation in S2 cells transfected with dsRNA chc. We also can hypothesize that in addition to clathrin-mediated endocytic pathway, another pathway can be used by FDP to enter S2 cells. This hypothesis was reinforced by the higher decrease of phytoplasma entry into S2 cells when cytochalasin D, which inhibits actin filament polymerisation, was added to S2 cells. Actin polymerisation is required for several endocytic pathway in non-phagocytic cells and in phagocytosis in phagocytic cells like S2 cells. Although caveolin-mediated endocytosis can be ruled out based on our results, non-clathrin/non-caveolin-dependent endocytosis cannot be excluded. Indeed, the dynein light chain 1 and tubulin beta were found among the proteins that preferentially interact with VmpA. They are implicated in the transport of many cargoes in the cell, including endosomes^41^. Nevertheless, few works of the role of dynein and tubulin in the first steps of endocytosis have been published. As example, they are implicated in the internalization of clathrin-independent vesicles that carry CD47 cargo in T cell^42^. Regarding to the concentrations used in the literature (between 10 and 50 µM^31,32^), we can also suggest that the concentrations of CPZ used in our experiments were not sufficient to totally inhibit clathrin mediated endocytosis in S2 cells. In the case of dengue virus 2, a dose dependent inhibition of virus infection of C6/36 mosquito cells was observed between 20 and 50 µM of chlorpromazine^32^. Our results also show that not all phytoplasmas in contact with S2 cells were colocalized with GFP-Clc. Recruitment of clathrin during bacterial entry is a dynamic and quick event that could explain these observations^43^ or could be representative of another route of entry in insect cells.

The pictures of Fig. 2 show that VmpA staining labelled the membrane of phytoplasmas that looked intact, suggesting that FDP could survive into S2 cells that are likely macrophage-like lineage. Phytoplasmas multiply in the haemolymph and can infect haemocytes^10,44^. Infection of haemocytes of its insect vector by phytoplasmas could be a way to spread into the insect and invade numerous tissues after the crossing of the midgut barrier. Such haemocyte infection has also been observed in vivo for *Spiroplasma kunkelii* in its vector *Dalbulu*s *maidis*^45^.

In our conditions, FDP did not enter the *E. variegatus* cells in culture with a sufficient efficiency to test different drugs inhibiting endocytic pathways. However, inhibition of the expression of clathrin heavy chain gene into *E. variegatus* decreased the colonization of the midgut and the salivary gland cells by phytoplasmas, suggesting that clathrin is implicated in the insect cell infection by the FDP and is consistent with the results obtained during S2 infections. It should also be noted that the effect of clathrin knockdown by RNAi on FD phytoplasma colonization is much stronger in salivary gland cells than in midguts. This could be explained by the detection of phytoplasmas in the intestinal lumen attached to the perimicrovillar membrane that could not cross epithelial cells due to the chc silencing. This detection of phytoplasmas would lead to underestimating the effect of RNAi on the crossing of the intestinal barrier by phytoplasmas. Similar differences between viral load in intestine and haemolymph have been observed for the tomato yellow leaf curl virus when its whitefly vector *Bemisia tabaci* ingested CPZ or chc dsRNA. The relative quantity of virus increased into the midgut while it decreased into the haemolymph^34^. Another hypothesis could be that clathrin is implicated in the entry into haemocytes and salivary gland cells, but should be less implicated to invade intestinal cells. Some pathogens use different endocytic mechanisms when they enter different cell types. This is the case of *Francisella* that is internalized via caveolin-mediated phagocytosis in phagocytes^46^ but invades hepatocytes using clathrin-mediated endocytosis, independently of caveolin^47^.

The adaptor protein complex AP-1 and AP-2 were detected among the proteins complexes that interacted with VmpA. The AP-2 protein is the most important adaptor protein complex for clathrin-mediated endocytosis and may also be implicated, with clathrin, in entry of FD phytoplasma into non-phagocytic insect cells as it is the case for FimH-mediated *E*. *coli* invasion of host cells and for *Francisella tularensis* to invade hepatocytes^28,47^. Usually, AP-1 is implicated in the assembly of clathrin lattices on the Golgi apparatus and in recycling endosomes in eukaryotic cells. However, colocalization and AP-1 knockdown by RNAi showed that AP-1 is implicated in the invasion of eukaryotic cells by *L*. *monocytogenes*^48^. Moreover, AP-1 was found to localize to the phagocytic cups of mammalian macrophages and the amoeba *Dictyostelium discoideum*^49^. We can hypothesize that AP-1 could be implicated in entry into phagocytic cells and AP-2 in non-phagocytic cells, but this remain to be elucidated.

Use of RNA interference to inhibit gene expression in *E. variegatus* has been demonstrated after microinjection of dsRNA^50^. The silencing of the two genes muscle actin and ATP synthase was observed up to 14 days, leading to almost complete silencing. So, we decided to use RNAi but we preferred the oral route to administer dsRNA and target the midgut. In our case ingestion of dsRNAs allowed survival rates comprised between 53 and 92% after 7 days on plant, which was superior to those observed by Abba and collaborators that were comprised between 0 and 50% after 3 days on plant^50^. The survival rate in our case was sufficient to test RNAi onto infection rate of *E. variegatus* by FD phytoplasmas. RNAi technology was also used through artificial feeding of dsRNA with the Cicadellidae *Psammotettix striatus* to knockdown the expression of α-tubulin^20^. The authors found a significant decrease of α-tubulin mRNA level in total RNA extracted from insects with levels similar to ours. In our study, we found that infection by phytoplasmas had no effect on the decrease of chc mRNA after ingestion of chc dsRNA. This suggests that FDP does not possess RNA silencing suppressor directed against the insect machinery as it is the case for the wheat blue dwarf phytoplasma whose SWP16 candidate effector suppress systemic RNA silencing in *Nicotiana benthamiana*^51^. We analysed RNA extracted from midguts and heads to see if a distal effect of the RNAi could be observed. As soon as 7 days, silencing of the target gene was observed both at the ingestion site, i.e. midgut, and in the head. Similar results has been observed by Galetto et col. when the insect have been injected with dsRNAs^52^.

After knockdown of the target gene α-tubulin using oral route, the transmission efficiency of WBD phytoplasma by *P. striatus* to wheat was reduce about twice but the phytoplasma amount in the insect was not mentioned^20^. Twenty-one days after injection of ATP synthase dsRNA and acquisition of FD phytoplasmas, the amount of phytoplasma 16SrRNA was 4 time lower than those measured in insects injected with GFP dsRNAs^52^. In our study, twenty-one days after ingestion of chc dsRNA and acquisition of phytoplasmas, the amount of tuf mRNAs of FD phytoplasmas was about 7 times lower in the midgut and 33 times lower in the salivary gland cells. This decrease of tuf mRNAs can be related to a decrease of phytoplasma number or to a decrease of physiologically active forms of the bacteria as it has been observed for the CY phytoplasma during the plant and insect infections^53^. If these reductions of phytoplasma titer are linked to a reduction of transmission by *E. variegatus* to plant remains to be elucidated.

Collectively, we showed that FD phytoplasma enters S2 cells using clathrin-mediated endocytosis at least and that clathrin is also required for full invasion of the insect vector *E. variegatus*. Our results are promising and RNAi could be a technique useful to test the suppression of phytoplasmas transmission by their insect vector.

## Methods

### Phytoplasmas, insect rearing and cell lines

The phytoplasma strain FD92 (FDP) was originally transmitted to broad bean by infected *S*. *titanus* sampled in FD-diseased vineyards in southwest France^54,55^. FDP was then continuously maintained in broad beans by *E. variegatus* transmission, as described by Caudwell et al.^9^.

Healthy *E. variegatus* leafhoppers originally collected in Villenave d’Ornon, France, were reared in cages on broad beans *Vicia faba* var. aquadulce and oats *Avena sativa* from seeds purchased from Castros Gerand and Jardiland, respectively, at 25 °C in green house under 16 h light/8 h dark photoperiod. To obtain infected *E. variegatus*, L4–5 nymphs were transferred by groups of 100 on FDP-infected broad bean for phytoplasma acquisition. One week later, *E. variegatus* were placed in cages on healthy broad bean for a latency period of 3–4 weeks before use. This study complies with relevant institutional, national, and international guidelines and legislation.

The *E. variegatus* Euva-12 cell line was established from embryos of *E. variegatus*, as previously described for Ciha-1 cells^56^. In brief, the eggs were sterilized with bleach solution and then with 70% ethanol. After rinsing, the eggs were ground in modified culture medium made of 350 mL Schneider’s Drosophila medium (Invitrogen), 100 mL Grace’s insect cell culture medium (Invitrogen), 50 mL heat-inactivated fetal bovine serum (Eurobio) and 2 mL G-5 supplement (Invitrogen). The Euva-12 cells were cultivated at 25 °C. After the first colonies developed and the cell line was established, the cells were passed using trypsinization every week with an additional change in the medium during the week. The *Drosophila melanogaster* S2 cells (Invitrogen) were cultured at 28 °C in Euva-12 culture medium and were passed twice a week.

### Pull-down assay and mass spectrometry analysis

The VmpA-His_6_ protein was purified as described previously^57^ on HIS-Select nickel affinity gel-packed columns (Sigma). Briefly, the nickel column was conditioned with 0.05 M sodium phosphate buffer at pH 7.4 with 0.2% Triton X-100. Imidazole elution concentrations were 0.25 M. The purification of protein was monitored by sodium dodecyl sulfate–polyacrylamide gel electrophoresis (SDS-PAGE), and Western blotting was applied with rabbit polyclonal antibodies (PAbs) raised against the His_6_-tagged recombinant VmpA (His_6_-VmpA) produced by Covalab (Villeurbanne, France).

Salivary gland cell proteins of *E. variegatus* were extracted from about 100 dissected salivary glands. Organs were homogenized in 400 µL of Rx buffer^15^ and centrifuged for 1 min at 13,000×*g*. The proteins contained in the supernatant were aliquoted and frozen before used. The recombinant VmpA-His_6_ was covalently linked to amine-modified magnetic beads (Dynabeads M-270 Amine, Invitrogen) according to the manufacturer’s instructions. The GFP protein served as control. Beads were then incubated with salivary gland proteins at room temperature for 1 h with tilting rotation and washed three times with PBS. The insect proteins bound to VmpA-beads, or GFP-beads, were eluted with hot Laemmli buffer and migrated on SDS-PAGE. Salivary gland proteins interacting with VmpA-His_6_ or GFP were excised from stained gel and digested with trypsin as previously described^58^. The resulting digestion was analyzed by liquid chromatography tandem mass spectrometry (LC–MS/MS) as routinely performed on the proteomics platform of the University of Bordeaux, France. SDS-PAGE was used for sample preparation and cleaning purpose. No fractionation was performed. A single band with complete proteome was cut for each sample. Search parameters were as follows: mass accuracy of the monoisotopic peptide precursor and peptide fragments was set to 10 ppm and 0.02 Da respectively. Only b- and y-ions were considered for mass calculation. Oxidation of methionines (+ 16 Da), phosphorylation of S, T and Y (+ 80 Da) and protein N-terminal acetylation (+ 42 Da) were considered as variable modifications while carbamidomethylation of cysteines (+ 57 Da) was considered as fixed modification. Two missed trypsin cleavages were allowed. Spectra from peptides higher than 5000 Da or lower than 350 Da were rejected. Peptide validation was performed using Percolator algorithm (1) and only “high confidence” peptides were retained corresponding to a 1% False Positive Rate at peptide level. Peaks were detected and integrated using the Minora algorithm embedded in Proteome Discoverer. Quantitative data were considered for proteins quantified by a minimum of two peptides and a statistical p-value lower than 0.05. Protein abundances were calculated as the sum of all unique peptide intensities. Protein ratios were calculated as the median of all possible pairwise peptide ratios for a given protein. Only unique peptides were considered for this calculation. Normalization was performed based on total peptide amount. Data were searched by SEQUEST through Proteome Discoverer 2.2 (Thermo Fisher Scientific Inc.) against a collection of peptide sequences originating from the conceptual translation of RNAseq data from *Euscelidius variegatus*. The dataset analyzed during the current study are available in the Transcriptome Shotgun Assembly database on NCBI: Euscelidius variegatus, transcriptome shotgun assembly repository, GenBank accession: GFTU00000000.1. After identification of the peptides produced by LC–MS/MS, only the proteins with a ratio VmpA/GFP superior to 1.50 and below 0.65 were kept to construct Fig. 1. The proteins that significantly interacted with VmpA or GFP were classified in function of the KEGG BRITE classification (www.kegg.jp/kegg/kegg1.html).

### Construction of S2 cells expressing GFP-clathrin tagged protein

The coding sequence of the clathrin light chain (clc) was amplified from the *E. variegatus* cDNA using sequence-specific primers listed in Table 3 and cloned into the Gateway vector pDONR (Invitrogen) in *Escherichia coli* Stellar competent cells (Takara Bio). Sequence of cloned fragments was verified by sequencing (Genewiz). LR recombination was performed with the plasmids pA-GFP-W (Carnegie) allowing the production of the recombinant proteins GFP-Clathrin using the actin promotor. To validate the subcellular localization of the GFP-Clc, the recombinant plasmids were isolated from *E. coli* and transferred into S2 cells using FuGENE HD Transfection Reagent (Promega) according to the manufacturer’s instructions. The S2 cells were cultured for 24 h before nuclei staining with DAPI (Sigma, 2 µg/mL) and microscopic observations (see below). To select stable transfected S2 cells, the blasticidine-S deaminase (*bsd*) gene that confers resistance to blasticidine from the pIB/V5-His_6_ DEST (Invitrogen) was integrated into the *Hpa*I restriction site of the pA-GFP-clc using the In-Fusion HD Cloning Kit (Takara Bio). The S2 cells were transfected as previously described with the pA-clc-GFP-bsd plasmid and cultured for 24 h. The medium was replaced by medium containing blasticidin (Thermo Fisher, 10 µg/mL) for three days, then the cells were cultivated in medium without antibiotic. The S2 cells expressing the fluorescent GFP-Clc were enriched using the BDFACSARIA (University of Bordeaux's cytometry platform).

**Table 3:** Sequence of primers used in this study.

Aplication	Primer name	5′–3′ sequence	Target gene	Species target/plasmid	Product size (n)	PCR efficiency (%)
dsRNA synthesis	S2-ClatHC-F3-T7	TAATACGACTCACTATAGGGGATGCGcGAACActTGGAgC	Clathrin heavy chain (chc)	*Drosophila melanogaster*	421	–
	S2-ClatHC-R3-T7	TAATACGACTCACTATAGGGGGTTATTGAGAGATTGGACTG				
	Ev-ClatHCH1-T7-F2	TAATACGACTCACTATAGGGGTGTTGTTGAGGGACTGGACA	Clathrin heavy chain	*Euscelidius variegatus*	429	–
	Ev-ClatHCH1-T7-R2	TAATACGACTCACTATAGGGGTGCGAGAACATCTGGAACTG				
	eGFP-ds-T7F1	TAATACGACTCACTATAGGGGCAAGGAGGACGGCAACATCC	GFP	pAGW	323	–
	eGFP-ds-T7R1	TAATACGACTCACTATAGGGGCGAACTCCAGCAGGACCATG				
Real-time PCR	S2-GST-F2	AAGTTGGTCACCCTGAATGC	Glutathione S-transferase (GST)	*Drosophila melanogaster*	180	107
	S2-GST-R2	TGGGTAGGGTTCCAACAG				
	S2-EF1-F2	AGGCAGGTATCTCGAAGAAC	Elongation factor 1 α1 (EF1)	*Drosophila melanogaster*	103	107
	S2-EF1-R2	GAGGAGTCCATCTTGTTCAC				
	S2-ClatHC-F5	CATGCCGATGAGCTAGAGGA	Clathrin heavy chain	*Drosophila melanogaster*	128	100
	S2-ClatHC-R4	GCTAATTCGGTGAACATTCCC				
	GST1_F257c^a^	CCAAGGACCCCAAGAAGCGA	Glutathione S-transferase	*Euscelidius variegatus*	113	96
	GST1_R369c^a^	TGGCGCTCCTCCAAACATCA				
	EvTubB_F1	CGCCCAGAGGTCTCAAGATG	Tubuline β	*Euscelidius variegatus*	148	94
	EvTubB_R2	ATCTCGTCCATGCCCTCGC				
	EvClatH1_F3	AGGCCACACATTTGAGCAAG	Clathrin heavy chain	*Euscelidius variegatus*	120	97
	EvClatH1_R1	GAAAGAGTTCCAGGGTGCAG				
	3Fbl^b^	TGAAGATCCAGTACGTGATTTAGAC	Elongation factor Tu (EFTu)	FD92 phytoplasma	158	96
	3R1^b^	TTTTAGTTTCTTTAATACCTATGATTTC				
Cloning	GFP_clc_attB1-F	GGGGACAAGTTTGTACAAAAAAGCAGGCTTCGACTTCGGAGACGATTTCG	Clathrin light chain	*Drosophila melanogaster*	745	–
	GFP_clc_attB2-R	GGGGACCACTTTGTACAAGAAAGCTGGGTCTTAGGCGAGTGCGTAATTAAAACTAC				
	pAG-bsd-F	GCAATTGTTGTTGTTAACTTGTTCCGAAGGGTTGTGTCAC	Blasticidin-S deaminase (bsd)	pIB/V5-His	650	–
	pAG-bsd-R	TGCAATAAACAAGTTAACCGGGACGTGTCAGTCCTGCT				

T7 promoter sequence is underlined.

^a^Primers were described in Galetto et al. (Galetto, L., Abbà, S., Rossi, M., Vallino, M., Pesando, M., Arricau-Bouvery, N., et al. (2018) Infect Immun 86: e00042–18).

^b^Primers were described in Eveillard et al. (Eveillard, S., Jollard, C., Labroussaa, F., Khalil, D., Perrin, M., Desqué, D., et al. (2016) Plant Biot Interact 1762).

### Infection of insect cells with phytoplasmas

Salivary glands of 20 FDP infected *E. variegatus* were dissected after the insects were anaesthetized with CO_2_ and sterilized with bleach solution for 3 min and then with 70% ethanol for 3 min. After three washes in sterile water, they were ground in 800 µL culture medium. Euva-12 cells of culture passages 48 to 57, depending on the assay, were cultivated on coverslips in 24-well plates (Falcon), as previously described^59^. S2 cells of culture passages 15 to 37 and transfected S2 cells expressing GFP-Clc were cultivated on coverslips in 24-well plates previously treated with poly-lysine (Sigma) according to the manufacturer’s instruction. The cells were infected with 100 µL of FDP preparation within 400 µL of culture cell containing 100 µg/mL penicillin and 1.25 µg/mL fungizone for one day to 21 days.

The endocytosis inhibitors chlorpromazine (Sigma), cytochalasin D (Sigma) and nystatin (Sigma) were added to S2 cells for 1 h and for all compounds two concentrations were tested (10 and 30 µM) in a final volume of 400 µL. The treated cells were then FDP infected with 100 µL as previously described, in the presence of the inhibitors. For each experiment, 20 fields were randomly observed per condition (see below), and three independent assays were performed. The viability of insect cells in presence of the endocytosis inhibitors was measured using 0.4% Trypan blue solution (GIBCO/BRL) for 3 min before cell observation with light microscope. The number of unstained and viable cells were compared to the number of stained and dead cells.

When the cells were transfected with dsRNA, they were infected with phytoplasmas seven days later. For each experiment, 15–20 fields were randomly observed per condition (see below), and five independent assays were performed and compiled.

### Immunofluorescent staining and microscopic observations

After incubation with FDP, the infected cells were fixed with 4% paraformaldehyde (Electron Microscopy Science) for 15 min and then washed 3 times in PBS. Next, the cells were incubated for 30 min with rabbit anti-VmpA polyclonal antibodies (diluted 1:7000, Covalab). After three washes, insect cells were incubated for 30 min with secondary Alexa 633-conjugated goat anti-rabbit IgG (Thermo Fisher Scientific) at a 1:200 dilution when an actin labelling was made with fluorescent phalloidin or with secondary Alexa 568-conjugated goat anti-rabbit IgG (Thermo Fisher Scientific) at a 1:200 dilution. This staining labelled external phytoplasmas. After three washes, the cells were fixed with 4% paraformaldehyde containing 0.2% Triton X-100 for 15 min, and then washed 3 times in PBS. The cells were incubated for 1 h with Alexa 568-phalloidin (Invitrogen) at a 1:40 dilution and secondary Alexa 488-conjugated goat anti-rabbit IgG (Thermo Fisher Scientific) at a 1:200 dilution. This staining labelled both external and intracellular phytoplasmas. After washing, the nuclei were stained for 5 min in water with DAPI (1 mg/mL, Sigma). The transfected S2 cells expressing GFP-Clc were fixed with paraformaldehyde and DAPI staining as previously described. The cells were mounted in ProLong Gold antifade reagent (Thermo Fisher Scientific) and were imaged using a Zeiss LSM 880 confocal laser-scanning microscope with an objective with a numerical aperture of 1.4. The resolution was improved with an Airyscan detector. Excitation wavelengths, which are sequentially individually switched on, are 405 nm, 488 nm, 561 nm and 633 nm for DAPI, Alexa 488, Alexa 568 and 633, respectively. The same emission filter (BP 420–460 nm + LP 500 nm) was used for the 4 channels.

When the S2 cells expressing the fluorescent GFP-Clc were infected with FDP, after 18 h the cells were fixed with paraformaldehyde and external phytoplasmas were labeled with rabbit anti-VmpA polyclonal antibodies and secondary Alexa 568-conjugated goat anti-rabbit IgG as previously described. The cells were imaged using a Zeiss LSM 880 confocal laser-scanning microscope with an objective with a numerical aperture of 1.4. Wavelengths 488 and 561 nm were used to excite respectively GFP and Alexa 568. In order to remove auto-fluorescence, the spectral acquisitions with a 32-channel linear detector for spectral analysis were achieved before online spectral unmixing based on reference spectra of GFP and Alexa 568.

When the cells were FDP infected in presence of the endocytosis inhibitors or after transfection with dsRNA, the external phytoplasmas were labeled as previously described with anti-VmpA polyclonal antibodies and secondary Alexa 488-conjugated goat anti-rabbit IgG and the internalized phytoplasma with secondary Alexa 568-conjugated goat anti-rabbit IgG. The cells were labeled with DAPI. Then, the cells were imaged using a Zeiss AxioImager epifluorescent microscope with an objective with a numerical aperture of 1.4. The filters used for excitations and emissions were BP 377/50 and BP 447/60 for DAPI, BP 472/30 and BP 420/35 for Alexa 488, and BP 562/40 and LP 593 for Alexa 568, respectively. For Z-stack acquisition, images were acquired every 0.2 µm from the bottom to the top to S2 cells and Z-projections were performed before counting of intracellular and extracellular phytoplasmas with the free software package Fiji of ImageJ-win64 (http://imagej.nih.gov/ij/)^60^.

### RNAi on S2 cells and *E. variegatus* insects

The clathrin heavy chain (chc) and GFP (control) sequences were amplified by PCR using sequence-specific primers conjugated with 20 bp of T7 RNA polymerase promotor (Table 3). The PCR products were utilized as templates for dsRNA synthesis using the HiScribe T7 High Yield RNA synthesis Kit (New England Biolabs). The precipitated dsRNAs were resuspended in 20 µL of RNAse free water. The purity and integrity of dsRNAs were determined using NanoVue Plus spectrophotometer (GE Healthcare) and migration on agarose gel electrophoresis. S2 cells were transfected with 2 µg dsRNA using FuGENE HD Transfection Reagent. After two and seven days the total RNA was extracted (see below).

Healthy young and synchronized adults were fed through a parafilm membrane during 2 days. To minimize the volume included in the cap of the 1.5-mL microtubes, we placed a 2 mm glass bead and 18 µL of a solution consisting of HEPES 8 mM, sucrose 280 mM, pH 7.4 plus 2 µg/µL of dsRNA between two parafilm membranes. After feeding, the insects were transferred to encaged FDP infected broad beans or non-infected plants for 7 days (100 insects per plant). Then, the insects were placed into non-infected broad beans for one to three weeks for incubation period that corresponds to 14, 21 and 30 days after the dsRNA ingestion. Midguts and heads were dissected after the insects were anaesthetized with CO_2_, and two organs of the same experimental condition were pooled and correspond to one sample. Four independent experiments were conducted and all results were mixed for analysis.

### mRNA analysis

The open reading frame of chc, GST and Tubβ were amplified from the *E. variegatus* cDNA, and chc, GST, Tubβ and EF1 using sequence-specific primers listed in Table 3 and cloned into the pGEM-T Easy Vector (Promega). Sequence of cloned fragments was verified by sequencing. These plasmids were used to verify that the efficiency of real time PCR was similar for all genes. The primers used for real time PCR were chosen outside the sequence used to synthetize the dsRNA.

Total RNA was extracted from S2 cells in culture 2 days and 7 days post transfection, and from dissected insects *E. variegatus* from which midguts and heads were separately analysed. In each experiment, two organs from two insects of the same condition were grouped in a same sample to be analysed. The RNA extraction was performed using TRIzol Reagent (Thermo Fisher Scientific) according to the manufacturer’s instructions. First-strand cDNA was synthetized using SuperScript III Reverse Transcripase (Thermo Fisher Scientific) and 2.5 µM Random hexamer primers (Invitrogen). Subsequently, real time PCR was performed on the LightCycler 480 Real-Time PCR system (Roche Diagnostics Corp) using sequence-specific primers listed in Table 3 and N′,N′-dimethyl-N-[4-[(E)-(3-methyl-1,3-benzothiazol-2-ylidene)methyl]-1-phenylquinolin-1-ium-2-yl]-N-propylpropane-1,3-diamine (SYBR) Green (Roche) according to the manufacturer’s instructions. The cycling program for all amplifications was as followed: 95 °C for 15 min, followed by 40 cycles of 95 °C for 30 s, 60 °C for 30 s and 72 °C for 30 s. The glutathione S-transferase (GST) and elongator factor 1 (EF1) served as reference genes for experiments with S2 cells, and glutathione S-transferase (GST) and tubulin β (Tubβ) served as reference genes for experiments with insects *E. variegatus*. The relative fold change in gene expression was calculated using the 2^−∆∆Ct^ method^61^ and by integrating the two reference genes^62^.

## Supplementary Information

Supplementary Table S1.

## Data Availability

The datasets used during the current study available from the corresponding author on reasonable request. The datasets analysed during the current study are available in the TSA: Euscelidius variegatus, transcriptome shotgun assembly repository, GenBank: GFTU00000000.1.
