# Supplementary material for: Flavescence dorée phytoplasma enters insect cells by a clathrin-mediated endocytosis allowing infection of its insect vector
Source: Sci Rep. 2023 Feb 7;13:2211. doi: 10.1038/s41598-023-29341-1 (PMC9905606; doi:10.1038/s41598-023-29341-1)
Supplement: Supplementary file 1 — Supplementary Table S1. [file 41598_2023_29341_MOESM1_ESM.pdf]

Table S1. Identification using BLASTP of the proteins interacting more with VmpA than with GFP or more with GFP than with VmpA after pull-down purification and LS-MS/MS analysis. The proteins that significantly interacted with VmpA or GFP were classified in function of the KEGG BRITE classification.

Protein (BLASTP)	Normalized abundance	Ratio VmpA/control	KEGG	KEGG BRITE
heat shock protein 90 [Graminella nigrifrons]	44 855 811	1,63	K09487	Chaperones and folding catalysts
protein disulfide-isomerase [Microplitis demolitor]	131 573 625	1,55	K13996	Chaperones and folding catalysts
Tubulin beta-1 chain OS=Notothenia neglecta GN=tubb1 PE=1 SV=1 - [TBB1_NOTNE]	60 307 834	1,59	K07375	Cytoskeleton proteins-Exosome
Tubulin beta-2 chain (Fragment) OS=Oomycete-like sp. (strain MacKay2000) GN=TUBB2 PE=2 SV=1 - [TBB2_OOMCK]	40 885 456	2,12	K07375	Cytoskeleton proteins-Exosome
Cystatin-A OS=Homo sapiens GN=CSTA PE=1 SV=1 - [CYTA_HUMAN]	1 852 265 889	107,67	K13907	Exosome
Barrier-to-autointegration factor OS=Homo sapiens GN=BANF1 PE=1 SV=1 - [BAF_HUMAN]	7 200 477	2,38	K21870	Genetic information processing/Chromosome and associated proteins
transcription factor BTF3 homolog 4 [Nilaparvata lugens] (NAC superfamily)	3 090 570	1,69	-	Genetic information processing/Transcription factors
Heterogeneous nuclear ribonucleoprotein Q OS=Homo sapiens GN=SYNCRIP PE=1 SV=2 - [HNRPQ_HUMAN]	6 209 618	1,77	K13160	Genetic information processing/Transcription
RNA polymerase II transcriptional coactivator (Bemisia) XP_018916495.1	10 524 683	3,27	-	Genetic information processing/Transcription
Serine/arginine-rich splicing factor 9 OS=Rattus norvegicus GN=Srsf9 PE=1 SV=1 - [SRSF9_RAT]	2 033 672	1,79	K21123	Genetic information processing/Transcription
Splicing factor U2af 38 kDa subunit OS=Drosophila melanogaster GN=U2af38 PE=1 SV=2 - [U2AF1_DROME]	3 550 386	2,93	K12836	Genetic information processing/Transcription
Splicing factor U2AF 50 kDa subunit OS=Drosophila melanogaster GN=U2af50 PE=1 SV=1 - [U2AF2_DROME]	2 228 868	2,47	K12837	Genetic information processing/Transcription
PREDICTED: testis-specific Y-encoded-like protein 2 isoform X1 [Hipposideros armiger] XP_019523705.1	44 059	5,00	-	Genetic information processing/Transcription
putative U5 small nuclear ribonucleoprotein 200 kDa helicase [Diaphorina citri]	1 020 982	2,37	K12852	Genetic information processing/Transcription
28S ribosomal protein S34, mitochondrial [Zootermopsis nevadensis]	5 361 461	1,80	K17412	Genetic information processing/Translation
30S ribosomal protein S19 OS=Sodalis glossinidius (strain morsitans) GN=rpsS PE=3 SV=1 - [RS19_SODGM]	4 548 811	107,00	K02966	Genetic information processing/Translation
39S ribosomal protein L54, mitochondrial [Trichogramma pretiosum]	11 467 756	1,50	K17435	Genetic information processing/Translation
40S ribosomal protein S4 OS=Lysiphlebus testaceipes GN=RpS4 PE=2 SV=1 - [RS4_LYSTE]	14 941 806	1,59	K02986	Genetic information processing/Translation
40S ribosomal protein S6 OS=Manduca sexta GN=RpS6 PE=2 SV=1 - [RS6_MANSE]	9 582 775	1,57	K02990	Genetic information processing/Translation
40S ribosomal protein S15 OS=Bos taurus GN=RPS15 PE=2 SV=3 - [RS15_BOVIN]	4 590 803	2,91	K02956	Genetic information processing/Translation
60S ribosomal protein L17 OS=Ixodes scapularis GN=RpL17 PE=2 SV=1 - [RL17_IXOSC]	15 306 064	1,60	K02879	Genetic information processing/Translation

Eukaryotic translation initiation factor 3 subunit D OS=Nasonia vitripennis PE=1 SV=1 - [EIF3D_NASVI]	18 308 894	1,88	K08860	Genetic information processing/Translation
importin-7 isoform X4 [Zootermopsis nevadensis]	3 282 083	2,20	K20223	Genetic information processing/Translation
Aspartate--tRNA ligase, cytoplasmic OS=Bos taurus GN=DARS PE=2 SV=1 - [SYDC_BOVIN]	40 759 470	1,52	-	Genetic information processing/Enzymes
Serine--tRNA ligase, cytoplasmic OS=Bos taurus GN=SARS PE=2 SV=3 - [SYSC_BOVIN]	556 534	2,38	-	Genetic information processing/Enzymes
AP-1 complex subunit beta-1 [Nilaparvata lugens]	151 310 055	1,51	K12392	Membrane trafficking
AP-2 complex subunit alpha-2 OS=Bos taurus GN=AP2A2 PE=1 SV=1 - [AP2A2_BOVIN]	8 578 434	1,85	K11824	Membrane trafficking
dynein light chain 1, cytoplasmic isoform X1 [Aedes aegypti]	1 382 766	1,51	K10416	Membrane trafficking
NcSP75 [Nephotettix cincticeps]	1 027 383	1,66	-	Membrane trafficking
Probable coatomer subunit beta' OS=Caenorhabditis elegans GN=copb-2 PE=3 SV=3 - [COPB2_CAEEL]	5 821 274	1,53	K17301	Membrane trafficking
DNA/RNA non-specific endonuclease [Bemisia tabaci]	6 798 533	1,64	-	Metabolism
prostatic acid phosphatase [Plutella xylostella]	5 115 096	1,73	-	Metabolism
NADH dehydrogenase [ubiquinone] 1 alpha subcomplex subunit 8 (DNA_pol3_alpha superfamily) [Zootermopsis nevadensis]	1 534 267	1,68	K03952	Metabolism/Energy metabolism
acyl carrier protein, mitochondrial isoform X3 [Drosophila miranda]	2 159 214	1,63	K02078	Metabolism/Lipid metabolism
Patatin-04/09 OS=Solanum tuberosum PE=2 SV=1 - [PAT04_SOLTU]	10 196 947	2,19	K16816	Metabolism/Lipid metabolism
prostaglandin E synthase 3 isoform X2 [Zootermopsis nevadensis]	1 089 836	1,70	K15730	Metabolism/Lipid metabolism
Ceruloplasmin OS=Ovis aries GN=CP PE=2 SV=1 - [CERU_SHEEP]	5 361 000	1,52	K13624	Metabolism/Metabolism of cofactors and vitamins
signal peptide peptidase-like 2B isoform X1 [Poecilia latipinna] - patellin-2-like isoform X2 [Branchiostoma belcheri]	437 125	1,94	K09597	Metabolism/Peptidases and inhibitors
PREDICTED: agrin [Neodiprion lecontei]	44 954 236	1,58	K06254	Proteoglycans
solute carrier family 35 member B1 (UAA transporter family) [Diaphorina citri]	1 180 193	1,76	K15275	Transporters
AN1-type zinc finger protein 4-like [Zootermopsis nevadensis]	12 576 619	1,83	K12163	Ubiquitin system
no BLAST sequence homology	1 314 400	2,05	-	No BLAST sequence
no BLAST sequence homology	439 883	2,05	-	No BLAST sequence
no BLAST sequence homology	59 674 231	1,79	-	No BLAST sequence
no BLAST sequence homology	4 664 797	1,74	-	No BLAST sequence
no BLAST sequence homology	4 172 489	1,72	-	No BLAST sequence
no BLAST sequence homology	1 210 832	1,69	-	No BLAST sequence
no BLAST sequence homology	3 290 241	1,58	-	No BLAST sequence
ervatamin-B-like [Arachis ipaensis]	1 047 217	0,42	-	Catabolic process

titin [Polistes dominula]	750 852	0,45	K12567	Cytoskeleton proteins
Myeloperoxidase OS=Homo sapiens GN=MPO PE=1 SV=1 - [PERM_HUMAN]	1 630 104	0,65	K10789	Exosome
translocon-associated protein subunit beta [Nilaparvata lugens]	7 337 917	0,65	K13250	Folding, sorting and degradation
Chromatin-remodeling complex ATPase chain Iswi OS=Drosophila melanogaster GN=Iswi PE=1 SV=1 - [ISWI_DROME]	329 438	0,62	K11665	Genetic information processing/Chromosome and associated proteins
Cytoplasmic FMR1-interacting protein OS=Drosophila melanogaster GN=Sra-1 PE=1 SV=1 - [CYFIP_DROME]	3 333 463	0,63	K05749	Genetic information processing/Messenger RNA biogenesis
Synaptic functional regulator FMR1 OS=Rattus norvegicus GN=Fmr1 PE=1 SV=2 - [FMR1_RAT]	52 844 430	0,64	K05749	Genetic information processing/Messenger RNA biogenesis
prohibitin-2 isoform X1 [Zootermopsis nevadensis]	23 580 328	0,67	K17081	Genetic information processing/Mitochondrial biogenesis
nucleolin isoform X2 [Peromyscus maniculatus bairdii]	12 200 153	0,66	K11294	Genetic information processing/Ribosome biogenesis
40S ribosomal protein S7 OS=Manduca sexta GN=RpS7 PE=2 SV=1 - [RS7_MANSE]	4 582 501	0,49	K02992	Genetic information processing/Translation
60S Ribosomal protein L36 (Pantala flavescens) (DNA_pol3_alpha superfamily)	4 510 346 738	0,36	K02919	Genetic information processing/Translation
Eukaryotic peptide chain release factor subunit 1 OS=Drosophila melanogaster GN=eRF1 PE=1 SV=2 - [ERF1_DROME]	20 234 799	0,64	K03265	Genetic information processing/Translation
elongation factor Tu, mitochondrial [Anopheles darlingi] Select seq gb|ETN59466.1|	2 773 741	0,61	K02358	Genetic information processing/Translation factors
Protein S100-A8 OS=Homo sapiens GN=S100A8 PE=1 SV=1 - [S10A8_HUMAN]	17 458 209	0,53	K21127	Immune system
Coatomer subunit beta OS=Gallus gallus GN=COPB1 PE=2 SV=1 - [COPB_CHICK]	19 127 132	0,67	K17301	Membrane trafficking
ER membrane protein complex subunit 4 [Nilaparvata lugens]	1 638 239	0,66	K23565	Membrane trafficking
probable pectinesterase 68 [Cajanus cajan] plante	10 586 010	0,62	K01051	Metabolism/Carbohydrate metabolism
NADH dehydrogenase [ubiquinone] 1 alpha subcomplex subunit 7 [Tribolium castaneum]	126 907	0,42	K03951	Metabolism/Energy metabolism
NADH dehydrogenase [ubiquinone] 1 subunit C2 [Ceratitis capitata]	14 598 355	0,58	K03968	Metabolism/Energy metabolism
short-chain specific acyl-CoA dehydrogenase, mitochondrial [Bemisia tabaci]	1 949 373	0,46	-	Metabolism/Lipid metabolism
Serpin B4 OS=Homo sapiens GN=SERPINB4 PE=1 SV=2 - [SPB4_HUMAN]	1 034 975	0,37	K13963	Metabolism/Peptidases and inhibitors
DDRGK domain-containing protein 1-like [Priapulus caudatus]	2 220 709	0,26	K23344	Ubiquitin system
ubiquitin-conjugating enzyme E2 N isoform X3 [Pseudomyrmex gracilis]	14 729 142	0,64	K10580	Ubiquitin system
FIT family protein CG10671 [Nilaparvata lugens]	960 223	0,65	-	Unknown
hypothetical protein MPLDJ20_60014 [Mesorhizobium plurifarium]	53 978 139	0,63	-	Unknown
hypothetical protein VOLCADRAFT_97128 [Volvox carteri f. nagariensis]	94 154 050	0,67	-	Unknown
no BLAST sequence homology	4 467 837	0,59	-	No BLAST sequence
